# Interplay of Epidermal Growth Factor Receptor and Signal Transducer and Activator of Transcription 3 in Prostate Cancer: Beyond Androgen Receptor Transactivation

**DOI:** 10.3390/cancers13143452

**Published:** 2021-07-09

**Authors:** Shian-Ren Lin, Hsiu-Lien Yeh, Yen-Nien Liu

**Affiliations:** 1 Graduate Institute of Cancer Biology and Drug Discovery, College of Medical Science and Technology, Taipei Medical University, Taipei 11031, Taiwan; d9813003@gms.ndhu.edu.tw; 2 General Education Development Center, Hsin Sheng Junior College of Medical Care and Management, Taoyuan 32544, Taiwan; qqhsiulien@gmail.com

**Keywords:** ADT, EGFR, STAT3, CRPC-Adeno, CRPC-NE

## Abstract

**Simple Summary:**

Androgen receptor (AR) signaling mainly controls prostate cancer (PCa) growth. Hence, the conventional regimen for PCa includes androgen deprivation therapy (ADT) or antiandrogen in order to reduce PCa recurrence. However, castration-resistant prostate cancer (CRPC) is insensitive to environmental androgen via self-supplementation of androgen or inactivation of AR signaling. Accordingly, CRPC has limited treating options and their outcome is poor. Previous studies reveal that forming CRPC needs to maintain cell growth under low AR signaling conditions and to mitigate AR dependency by differentiation. Previous studies show that the epidermal growth factor (EGFR) and signal transducer and activator of transcription 3 (STAT3) participate in the maintenance of PCa growth under androgen ablation. We have found a novel mechanism in which EGFR cooperates with STAT3 and initiates neuroendocrine differentiation. This review aims to summarize the recent findings on EGFR and STAT3 in CRPC induction and discuss the unsolved issues therein.

**Abstract:**

Prostate cancer (PCa) is one of the most common cancers in the world and causes thousands of deaths every year. Conventional therapy for PCa includes surgery and androgen deprivation therapy (ADT). However, about 10–20% of all PCa cases relapse; there is also the further development of castration resistant adenocarcinoma (CRPC-Adeno) or neuroendocrine (NE) PCa (CRPC-NE). Due to their androgen-insensitive properties, both CRPC-Adeno and CRPC-NE have limited therapeutic options. Accordingly, this study reveals the inductive mechanisms of CRPC (for both CRPC-Adeno and CRPC-NE) and fulfils an urgent need for the treatment of PCa patients. Although previous studies have illustrated the emerging roles of epidermal growth factor receptors (EGFR), signal transducer, and activator of transcription 3 (STAT3) signaling in the development of CRPC, the regulatory mechanisms of this interaction between EGFR and STAT3 is still unclear. Our recent studies have shown that crosstalk between EGFR and STAT3 is critical for NE differentiation of PCa. In this review, we have collected recent findings with regard to the involvement of EGFR and STAT3 in malignancy progression and discussed their interactions during the development of therapeutic resistance for PCa.

## 1. Introduction

Prostate cancer (PCa) is a leading cause of new cancer cases in men, which accounts for 6.8% of all cancer deaths and 14.1% of new deaths [1]. Low-risk (in situ carcinoma, low grade, or low metastatic potency) PCa patients usually do not need active treatment [2]. Intermediate- or high-risk cancer patients are usually prescribed a radical prostatectomy (RP) and radiotherapy (RT) prior to androgen deprivation therapy (ADT), which reduces the serum androgen content through a bilateral orchiectomy or luteinizing hormone-releasing hormone (LHRH) agonist based on the need for androgen for PCa growth. Androgen receptor (AR) signaling inhibitors (ARSIs) include nonsteroidal antiandrogens, with synthetic androstanes avoiding recurrences [3,4,5]. Unfortunately, about 10–20% of PCa cases recurred and developed into the castration-resistant phenotype of PCa (CRPC), recurrent tumors of which are insensitive to ADT [6]. According to their cellular characteristics, CRPC can be divided into two subgroups: adenocarcinoma (Adeno)-type CRPC (CRPC-Adeno, accounting for 80% of CRPC cases), which retain adenocarcinoma cell markers and cellular characteristics; and neuroendocrine (NE)-type CRPC (CRPC-NE), which exhibits NE cell characteristics and expresses NE-specific protein markers (n-Myc (*MYCN*) such as chromogranin A (*CHGA*), synaptophysin (*SYP*), and γ-enolase (*ENO2*)) instead of AR response genes (prostate-specific antigen (*PSA*)/*KLK3*, prostate-specific membrane antigen (PSMA)/*FOLH1*, SAM-pointed domain containing ETS transcription factor (*SPDEF*), and NK3 Homeobox 1 (*NKX3-1*)) [7,8,9]. CRPC-Adeno and CRPC-NE are aggressive phenotypes that offer limited therapeutic options such that they have poor prognoses when compared to the overall PCa population [10,11,12,13]. Accordingly, understanding the mechanisms of CRPC-Adeno and CRPC-NE development are urgently needed to develop better PCa therapies.

In order to overcome androgen dependency, PCa cells undergo several signaling changes that are grouped into the following strategies: 1. enhancing cell survival and growth; 2. mitigating androgen dependency; and 3. undergoing the epithelial-to-mesenchymal transition (EMT) and further NE differentiation (NED) [14]. To overcome androgen dependency, autocrine/paracrine androgen biosynthesis in PCa cells is activated to complement systemic androgen depletion [15]. Alternative splicing of the AR is also observed in CRPC cells that produce various AR variants [16]. The ligand-binding domain of AR variants is truncated into some constitutively active variants that provide growth signals even in androgen-ablated conditions [17]. In addition, the glucocorticoid receptor (GR) was found to be overexpressed in CRPC cells, which helped CRPC cells bypass AR signaling [18]. Particularly noteworthy is the fact that *de novo* CRPC-NE (CRPC-NE derived from NE cells) only occurred in approximately 1% of PCa cases and about 20% of CRPC cases [8,19], in which CRPC-NE development was partially induced by PCa treatment. Accordingly, the mechanisms of therapy-induced CRPC-NE development is critical for aggressive PCa, but a complete understanding remains unclear. Two hypotheses have been proposed for CRPC-NE development. The first is a hierarchical model that is based on the heterogeneity of PCa tumors in which ADT-sensitive cells were depleted during ADT, and whose recurrent tumors are mainly composed of ADT-insensitive CRPC or CRPC-NE cells. The second is the dynamic transdifferentiation model, which describes how ADT-sensitive adenocarcinoma cells are transdifferentiated into cancer stem cells (CSCs), followed by NED or are directly differentiated into CRPC-NE cells [20]. Both models explain CRPC-NE development as epigenetic modulations (alternative splicing of survival factors, tumor-suppressor gene mutations and silencing, or DNA methylation) that fit the hierarchical model and lineage plasticity in order to fit the other model [14,21,22]. Nevertheless, survival signals and EMT-related gene functions are altered, which are critical for CRPC-NE development.

**Figure 1:**
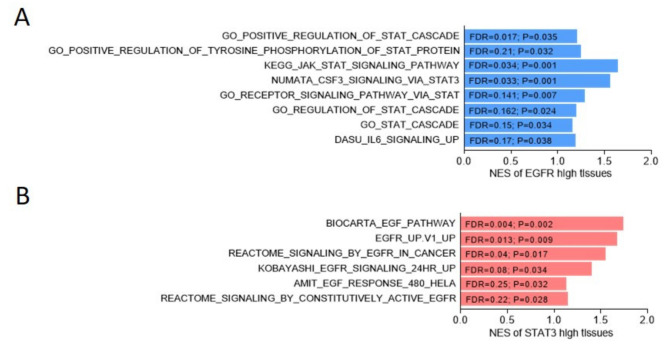
Positive association between EGFR and STAT cascade response pathways, or between STAT3 and EGFR-related signaling in the Cancer Genome Atlas (TCGA) prostate cancer dataset. (**A**) The gene set enrichment analysis (GSEA) of the TCGA prostate cancer dataset [31] showed the enrichment of EGFR (**A**) or STAT3 (**B**) expression among gene sets, the expression levels of which were upregulated in association with gene signature response to STAT cascade (**A**), Gene Ontology, KEGG, NUMATA [32], DASU [33], or EGFR-related signaling ((**B**), BIOCARTA, Broad Institute, REACTOME, and AMIT [34]). NES, normalized enrichment score. FDR, false discovery rate.

Epidermal growth factor (EGF) receptor (EGFR, also called Erb-b2 receptor tyrosine kinase 1, ErbB1) signaling controls various physiological roles from the cellular level to the organ level, such as organ development and wound healing [23]. In pathophysiological studies, aberrations (constitutive activation or overexpression) of EGFR signaling (or for EGFR itself) have often attracted attention, especially in tumorigenesis in various organs such as the lungs, breast, and colon [24,25,26]. The signal transducer and activator of transcription 3 (STAT3) are transcription factors that belong to cytokine receptor signaling, which is widely known to regulate immune responses [27]. In tumorigenesis, STAT3 is highly involved in immune checkpoints that maintain tolerance in the immune system against cancer cells [28]. Moreover, STAT3 participates in the EMT during tumor development and is associated with chemoresistance [29]. Through the analysis of upregulated genes during castration resistance, EGFR and STAT3 are positively correlated with PCa progression (Figure 1). That is, the EGFR and STAT3 play critical roles in forming castration resistance [30], but their interplay has not been extensively discussed in the literature. Recently, we discovered a novel crosstalk between EGFR and STAT3, which contributes to CRPC-NE development. Accordingly, this study summarizes recent knowledge about EGFR and STAT3 in CRPC and CRPC-NE development, in stand-alone as well as in crosstalk modes, which elicits complex questions that need to be answered.

## 2. EGFR Signaling

### 2.1. EGFR Signaling for Cell Survival/Growth under ADT

**Figure 2:**
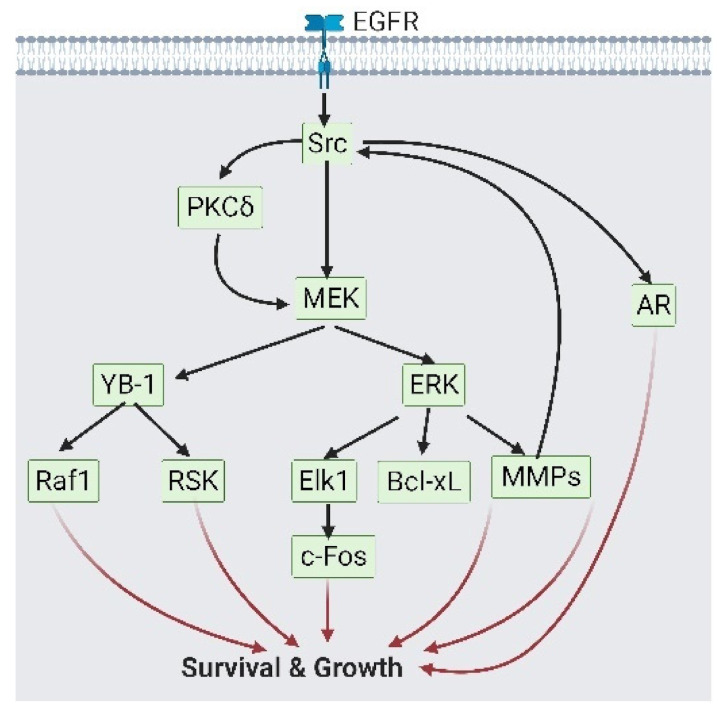
Epidermal growth factor receptor (EGFR) participates in cell survival via Src/ mitogen-activated protein kinase (MEK) signaling. As growth signaling from androgen receptor (AR) is reduced, EGFR on prostate cancer cells would phosphorylate Src. Src could activate both AR signaling and MEK/ERK or MEK/Y-box binding protein 1 (YB-1) signaling, which activate growth-related transcription factors. This figure is a summary of reference [36,37,38,39,40,41,42]. A black solid arrow represents signal transduction; a red arrow represents cell fate.

**Figure 3:**
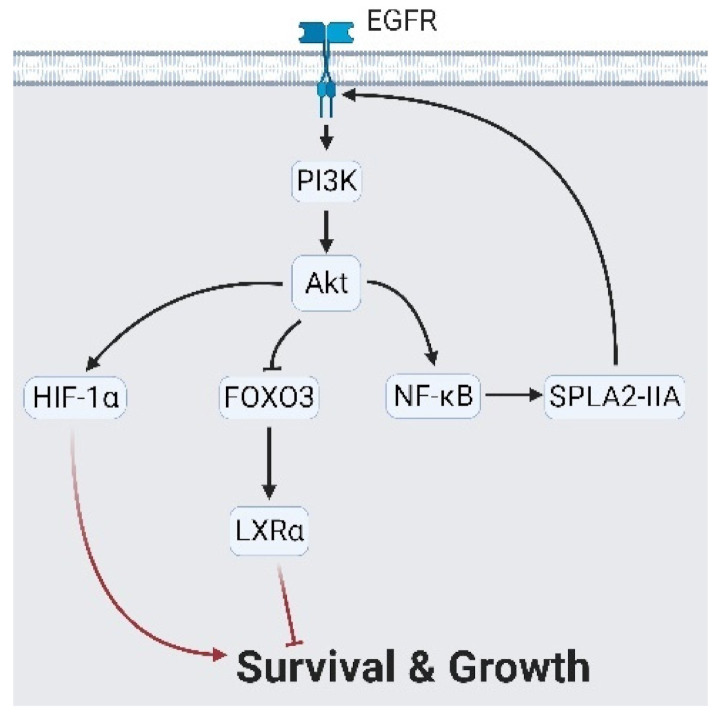
Epidermal growth factor receptor (EGFR) controls cell growth via Akt signaling. Akt signaling activates hypoxia-inducing factor 1α (HIF-1α) and forkhead box O3 (FOXO3)/ nuclear receptor subfamily 1 group H member 3 (LXRα) to produce a growth signal. Likewise, Akt stimulates nuclear factor kappa B (NF-κB) followed by secreted phospholipase A2-IIa (sPLA2-IIA), which reciprocally reinforces EGFR signaling. A black arrow represents signal transduction; a red arrow represents cell fate.

As described in previous studies, PCa cells activate complementary growth signaling (GR and EGFR) and AR alternative splicing to overcome the absence of AR signaling in an ADT condition [18,35]. The EGFR maintains cell survival by the following main cascades: The SRC proto-oncogene, non-receptor tyrosine kinase (Src)/mitogen-activated protein kinase (MAPK) cascade, and phosphatidylinositol 3-kinase (PI3K)/AKT signaling. Src phosphorylates MAPK kinase (MEK) with/without the paxillin (PXN) intermediate, followed by MAPK1 (also named extracellular signal-regulated kinase (ERK)) activation to upregulate pro-survival factors, such as BCL2-like 1 (Bcl-xL), matrix metalloproteases (MMPs), and Elk1/c-Fos [36,37,38,39,40]. Meanwhile, MEK phosphorylates Y-box-binding protein 1 (YB-1), which collaborates with ribosomal S6 kinase (RSK) and Raf-1 for the survival of gene expressions [41,42] (Figure 2). The second route of EGFR-related survival is through PI3K/AKT signaling. AKT acts via two axes: stimulating the nuclear factor (NF)-κB that triggers secretory phospholipase A2-IIa (sPLA2-IIa) expression and secretion; and the modulation of hypoxia-inducing factors (HIF)-1α and forkhead box O3 (FOXO3) expression levels to reduce liver X receptor alpha (LXRα) [43,44,45,46,47] (Figure 3). Collectively, EGFR signaling maintains cell survival under ADT and maintains expressions of pro-survival factors.

**Figure 4:**
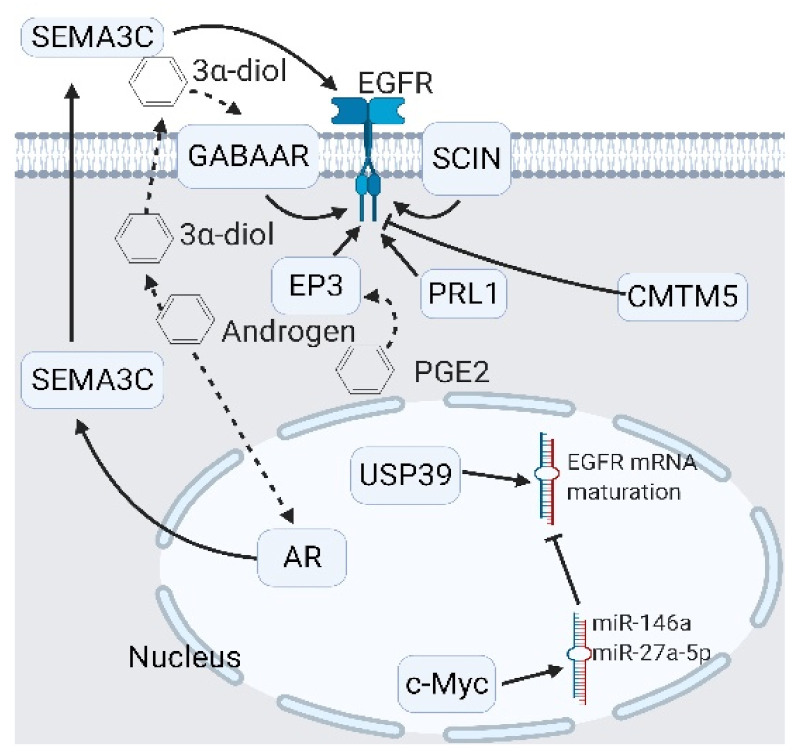
Upstream modulators of epidermal growth factor receptor (EGFR) signaling. Modulators of EGFR include extracellular modulators like semaphorin 3C (SEMA3C) and the androgen derivative 3α-diol, and intracellular modulators like prostaglandin E receptor 3 (EP3), scinderin (SCIN), and ubiquitin-specific peptidase 39 (USP39). Two inhibitors—the CKLF-like MARVEL transmembrane domain-containing 5 (CMTM5) and the phosphatase of regenerating liver 1 (PRL-1)—are identified. In particular, USP39 promotes EGFR signaling by promoting EGFR mRNA maturation. A black arrow represents signal transduction; a black dashed arrow represents translocated small molecules.

**Figure 5:**
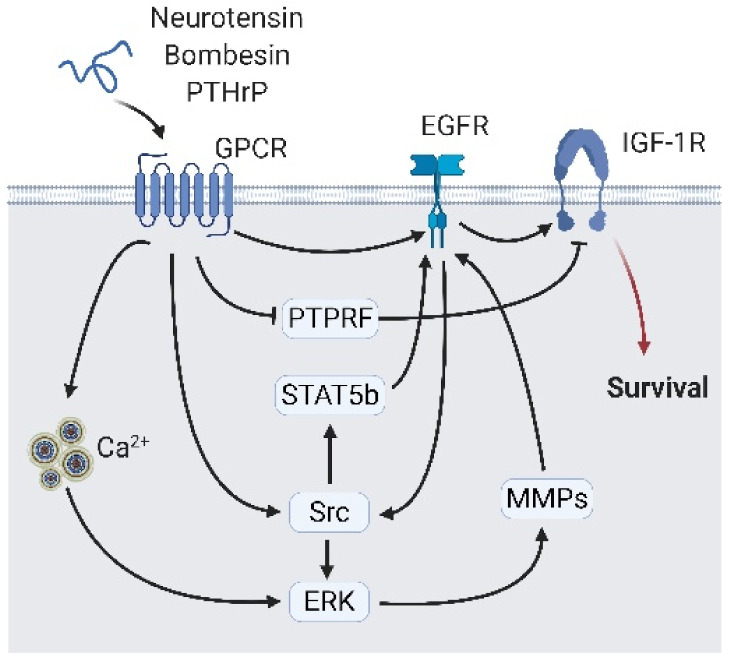
Neuropeptide secreted from neighbor neuroendocrine (NE) cells interact with epidermal growth factor receptor (EGFR) signaling. Peptide hormones including neurotensin, bombesin, and parathyroid hormone-related protein (PTHrP) bind to their receptor and interact with EGFR via direct interaction or indirect interaction, including calcium flux/ERK/Src or matrix metalloproteases, and Src/signal transducer and activator of transcription 5B (STAT5b). The EGFR transfer signals to the insulin-like growth factor 1 receptor (IGF-1R) and activates growth-related signaling. A black arrow represents signal transduction; a black faded arrow represents peptide hormones binding to the receptor; a red arrow represents cell fate.

In the upstream of EGFR signaling, several transducers were identified, including the scinderin (SCIN)/ubiquitin-specific peptidase 39 (USP39)/protein-tyrosine-phosphatase of regenerating liver 1 (PRL1) as activators and microRNA (miR)-146a/miR-27a-5p/CKLF-like MARVEL transmembrane domain containing 5 (CMTM5) as inhibitors [37,48,49,50,51] (Figure 4). During ADT, the EGFR upregulates its coactivators, ErbB2 and ErbB3, which promote androgen-sensitive cell survival under castration conditions [52]. In addition, the AR also promotes EGFR signaling through the upregulation of semaphorin 3C (SEMA3C), as mediated by plexin B1 [53]. Plexin B1 triggers phosphorylate AR at serine 81 to augment its stability [54]. Worthy of note is intraprostatic androgen production, which is an important factor in the induction of castration resistance [55]. Intraprostatic androgen is altered into 3α-androstanediol (also called 5α-androstane-3α or 17β-diol/3α-diol) and stimulates γ-aminobutyric acid receptor α (GABAAR) activity to promote the EGFR [56]. Fernández-Martínez and Lucio-Cazaña reported that intracellular prostaglandin E2 (PGE2) and its receptor, the EP2 prostanoid receptor, act as EGFR promoters, which are known as proinflammatory cytokines [45,57]. Prostate-specific membrane antigens (PSMA), also known as glutamate carboxypeptidase II (GCPII), are membrane-bound enzymes with unknown biological functions [58]. Perico et al. reported that PSMA formed a complex with filamin A, β1 integrin, p130CAS, c-Src, and the EGFR that phosphorylates the EGFR through β1 integrin/c-Src cascades [59]. Interestingly, NE cells in PCa, whether benign or malignant, secrete various neuropeptides into the tumor microenvironment (TME) [60,61]. DaSilva et al. demonstrated that several NE peptides such as neurotensin, a gastrin-releasing hormone, and the parathyroid hormone-related protein (PTHrP) transactivate EGFR signaling through intracellular calcium release and Src activation; in turn, the activated EGFR stimulates the insulin-like growth factor-1 receptor (IGF-1R) for survival [62,63,64,65] (Figure 5). These reports have partially revealed that the function of NE peptides in prostate tissues might be a survival signal. The description above emphasizes the impact of EGFR signaling in maintaining PCa growth under ADT (Figure 2, Figure 3, Figure 4 and Figure 5). Subsequently, we discuss the role of the EGFR in androgen independency.

### 2.2. EGFR Signaling Is Involved in Systemic Androgen Dependency

**Figure 6:**
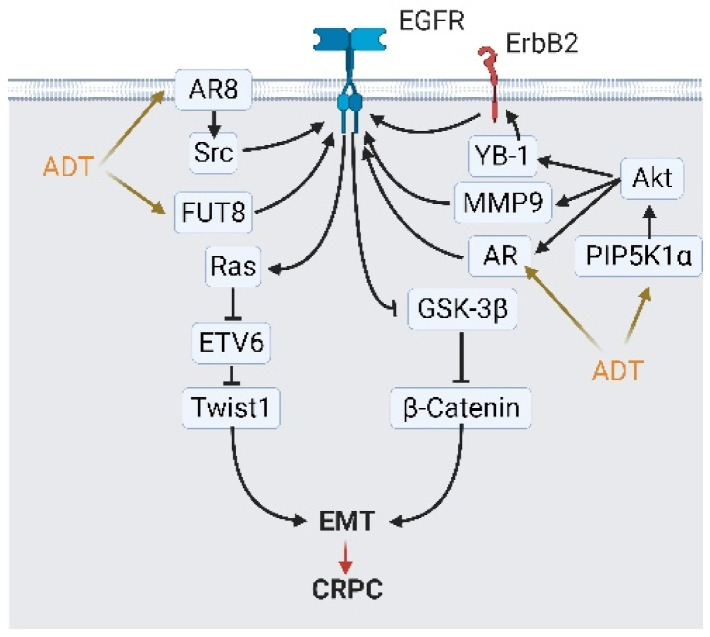
Androgen deprivation therapy (ADT) modulates epidermal growth factor receptor (EGFR) signaling and activates epithelial-to-mesenchymal transition (EMT) to escape from ADT. ADT modulates EGFR signaling and initiates EMT via four mediators: androgen receptor (AR) variants AR8, fucosyltransferase 8 (FUT8), AR overexpression, and phosphatidylinositol-4-phosphate 5-kinase type 1 alpha (PIP5K1A)/Akt signaling. Stimulated EGFR activate β-catenin and Ras signaling, which augments the expression of EMT-related transcription factor TWIST1 and others. A black arrow represents signal transduction; a red arrow represents cell fate; an orange arrow represents stimulations from ADT.

**Figure 7:**
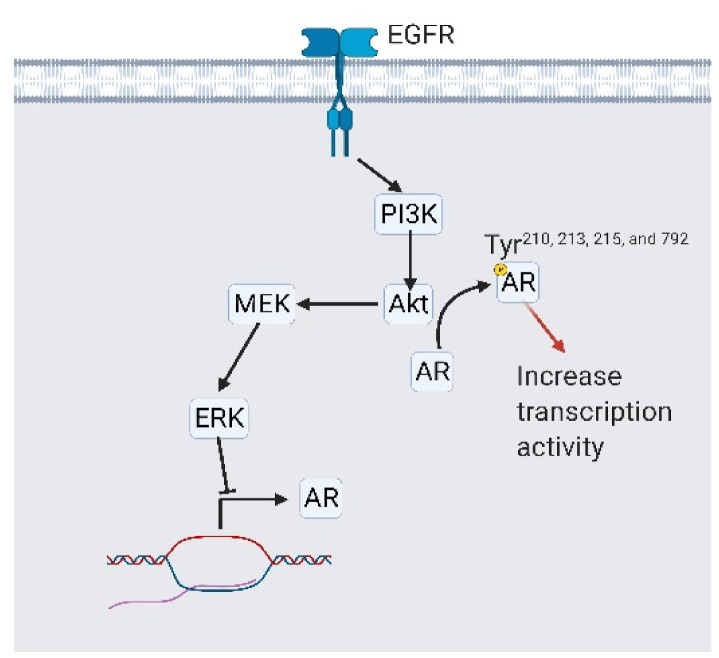
Epidermal growth factor receptor (EGFR) signaling is involved in systemic androgen receptor (AR) independency. EGFR activates PI3K/Akt/MEK/ERK signaling to reduce AR expression. On the other side, activated Akt phosphorylates AR at tyrosine 210, 213, 215, and 792, which stabilizes AR and promotes its transcriptional activity. A black arrow represents signal transduction; a red arrow represents cell fate.

The second aim of the EGFR in CRPC-Adeno and CRPC-NE induction involves androgen independency. Androgen independency is caused by the constitutive activation of intracellular androgen signaling (via AR overexpression, mutation, intracellular production of androgen, or transactivation) or the repression of AR expressions [66]. As androgen independency forms, ADT is no longer useful [66]. The EMT is necessary to repress the AR. It is mainly controlled by Wnt/β-catenin signaling [67,68]. The EGFR phosphorylates β-catenin through PI3K/AKT/heat shock protein family B (small) member 1 (HSPB1, previously termed HSP27) signaling that phosphorylates β-catenin, which initiates expressions of the EMT marker (vimentin, fibronectin, and snail family transcriptional repressor 2/SNAI2) [69]. Another pathway for the EGFR-triggered EMT is through Ras signaling. Ras inhibits ETS variant transcription factor 6 (ETV6) expression and represses twist family bHLH transcription factor 1 (TWIST1, another EMT marker) expressions [70] (Figure 6). In addition to triggering the EMT, EGFR signaling directly modulates AR expression. McAllister et al. discovered that EGFR/PI3K/AKT signaling directly phosphorylates AR at several serine sites including 210, 213, 215, and 792, which augments AR stability and transcription activity [54,71]. On the contrary, as EGFR/PI3K/AKT signaling alters downstream mediators of MAPK7 (termed MEK)/ERK signaling, AR expression is diminished [72] (Figure 7).

Upstream from EGFR, several activators, including fucosyltransferase 8 (FUT8), YB-1/ErbB2, and MMP9, stimulated EGFR signaling and pushed PCa cells into becoming androgen-independent [40,73]. During ADT, AR might be spliced into AR8, a membrane-bound variant. This membrane-bound variant acts as an anchor that brings the EGFR, Src, and AR together and is easily stimulated by the EGF [74]. Phosphatidylinositol-4-phosphate (PI4P) 5-kinase, type 1 alpha (PIP5K1α), a lipid kinase, phosphorylates PI4P into phosphatidylinositol 4,5-bisphosphate (PIP2), which is upregulated in high-grade PCa through an unknown mechanism [75]. Elevated PIP5K1α promotes AKT activity and subsequently triggers the EGFR via the modulation of the AR and MMP9 [40] (Figure 6 and Figure 7). The description above is the second map that demonstrates what the EGFR does in CRPC-Adeno and CRPC-NE induction.

### 2.3. Nuclear Translocation of the EGFR

The EGFR is distributed in plasma membranes in order to bind with environmental EGF signals [76]. In non-small-cell lung cancer (NSCLC), breast cancer, and colorectal cancer (CRC), the EGFR was also found to be expressed in nuclei [77,78,79]. Internalized EGFR becomes a transcriptional cofactor that reacts with STAT3, STAT5, and pyruvate kinase M2 (PKM2) to promote cell growth, migration, and the EMT [80,81,82]. The shuttling of EGFR to the nuclei may be mediated by clathrin-dependent or -independent endocytosis and a reaction with importin β1 [83,84]. Known triggers or enhancers of EGFR translocation include microsomal PGE synthase-1-mediated PGE2 synthesis, EGF-mediated Fas/YES proto-oncogene 1 (YES-1)/EGFR complex, followed by Fas phosphorylation at tyrosine 294 and dual oxidase 1 (DUOX1)-triggered hydrogen peroxide production [80,84,85,86]. Tan et al. summarized that cellular stress might contribute to EGFR translocation [87]. Tomas et al. further explored how stress-induced EGFR translocation is activated by p38/MAPK signaling [88]. In PCa, EGFR translocation is triggered by the internalization of extracellular vesicles or ADT, which can be blocked by estrogen receptor β activation [89,90]. However, the detailed mechanism of EGFR translocation in PCa has not been fully elucidated.

### 2.4. EGFR Signaling in NED

**Figure 8:**
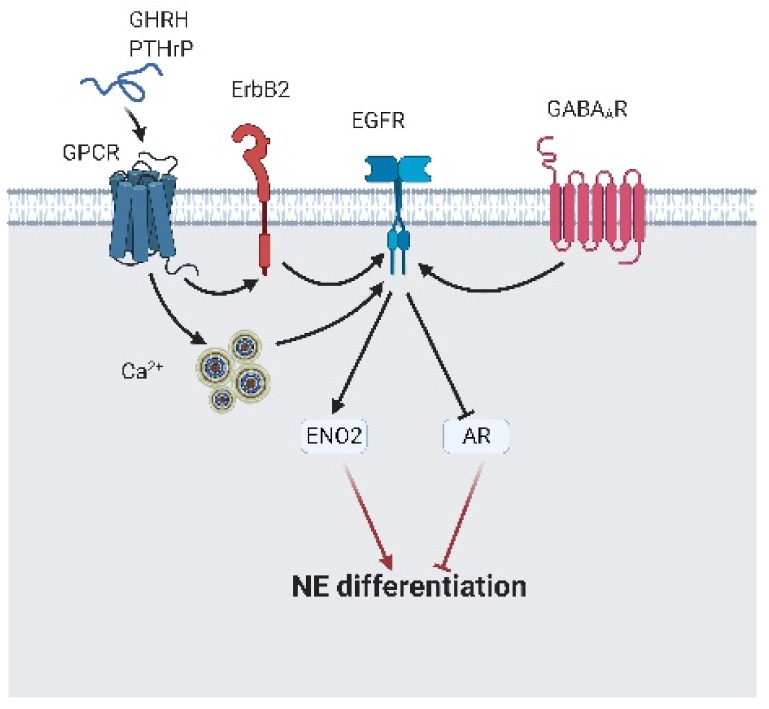
The epidermal growth factor receptor (EGFR) leads to neuroendocrine differentiation. γ-aminobutyric acid A receptor (GABAAR) directly interacts with EGFR and promotes neuroendocrine differentiation. Peptide hormone-like gonadotropin-releasing hormone (GHRH) and parathyroid hormone-related protein (PTHrP) bind to their specific receptor and indirectly activate EGFR via calcium flux or ErbB2. Activated EGFR induces γ-enolase (ENO2) expression and block androgen receptor (AR) expression, which results in neuroendocrine differentiation initiation. A black arrow represents signal transduction; a black faded arrow indicates peptide hormones that bind to the receptor; a red arrow represents cell fate.

NED is a complex progression in which cellular reprogramming and epigenetic modulation are involved [14,91,92]. EGFR signaling induces NED via the following actions: repressing AR expression and initiating ENO2 expression, which are activated by GABAAR, heparin-binding EGF-like growth factor (HB-EGF), PTHrP autocrine/paracrine; or growth hormone-releasing hormone (GHRH)-mediated calcium flux [63,93,94,95,96,97,98] (Figure 8). Interestingly, Martin-Orozco et al. reported that EGFR-mediated NED is activated via the PI3K/AKT/ERK cascade. Instead, this signaling axis reduced NED and promoted survival [99]. The molecular switch that guided EGFR signals away from the PI3K/AKT/ERK cascade to initiate NED is still covered. Work on EGFR signaling in CRPC-Adeno and CRPC-NE development has mainly focused on maintaining survival under castration conditions.

In addition to the above-mentioned mechanism, EGFR mutations are also thought to participate in NED; they are not mentioned in CRPC-NE development. Miyoshi et al. and Baglivo et al. reported that activated mutations for EGFR were found in large-cell NE lung cancer tissues, which helped lung adenocarcinoma cells acquire resistance to EGFR tyrosine kinase inhibitors (TKIs) and NED [100,101]. The exact mechanism of mutated EGFR-mediated NED is still unknown; however, the following mechanisms might contribute: the EGFR-mediated EMT and ectopic EGFR expression. Mutations at the tyrosine kinase domain of the EGFR could constitutively activate the EGFR without ligand binding, which further triggers lineage plasticity [21,102]. However, Niederst et al. reported that EGFR mutations in lung adenocarcinomas transformed small-cell lung carcinoma exhibiting NE cell markers but with a downregulated EGFR expression, and NED could not be attributed to EGFR mutations [103]. Ectopic EGFR expression could be attributed to membrane-to-nucleus trafficking, or from the exhibition of a nuclear translocation signal (NLS) caused by a mutation [82,104]. Nuclear EGFR cooperates with STAT3 and promotes inducible nitric oxide synthase (iNOS) expression, which has been reported to promote breast cancer metastasis [82]. EGFR mutations were identified in about 13% of PCa patients [105]. It is reasonable to consider EGFR mutations in NED, at least in part. Characteristics of EGFR-involved NED are of interest and present challenges when studying CRPC-NE.

## 3. Janus Kinase (JAK)/STAT3 Signaling in CRPC-Adeno and CRPC-NE

The JAK/STAT3 signaling cascade is an intracellular signaling mediator of cytokines and interferons that is a part of pathogen-associated molecular patterns (PAMPs) [106]. Immune responses to immune escape and immunotherapy have received more attention from researchers than other issues [28,107]. In addition to immune escape and immunotherapy, JAK/STAT3 (or IL-6/STAT3) signaling was also involved in the EMT and stemness [29]. Accordingly, in CRPC-Adeno and CRPC-NE induction, the role of STAT3 in the EMT or NED in PCa development was apparent.

Due to EMT-related transcription factors (SNAI1/2 and TWIST1) being the targets of STAT3, the activation of JAK/STAT3 signaling in cancer cells is commonly believed to initiate the EMT [29,108]. STAT3 received signals from various sources like cyclooxygenase 2 (COX2)-mediated PGE2 [109], IGF-1R [110], and enzalutamide-induced transforming growth factor (TGF)-β1 activation or AR repression [111], ATM serine/threonine kinase (ATM) [112], or ADT-mediated HSPB1 upregulation [113], which forms lipid rafts [114] and initiates expressions of EMT-associated genes including *twist* [110], the Pim-1 proto-oncogene, serine/threonine kinase (*pim-1*) [115], the PTTG1 regulator of sister chromatid separation, securin (*PTTG1*) [116], valosin-containing protein (*VCP*) [115], programmed cell death ligand 1 (*PD-L1*) [112], and *clusterin* [110]. Interestingly, Handle et al. reported that enzalutamide promoted JAK/STAT3 signaling through the blocking of the AR, which inhibits JAK expression in AR-positive PCa cells [117]. Activated JAK/STAT3 upregulates the expression of SOCS3 (a suppressor of cytokine signaling) in the presence of interleukin (IL)-6 and forms a negative feedback loop with STAT3, which further promotes the EMT instead of cell survival [118,119,120]. This JAK/STAT3/SOCS3 cascade acts inversely in AR-negative PCa cells; SOCS3 is an important survival factor in AR-negative PCa cells [121]. The previous literature on the survival role of SOCS3 has implied that AR might be a transcription factor for survival factors and a signaling modulator by switching signaling pathways from one direction to another in order to achieve inverse outcomes.

When involved in NED, STAT3 is no longer activated by JAK. Zinc finger and BTB domain containing 46 (ZBTB46)/leukemia inhibitory factor (LIF) signaling, pigment epithelium-derived factor (PEDF)/Ras homolog family member A (RHOA)/inhibitor of κB kinase (IKK)/NF-κB/IL-6, and LncRNA-p21/polycomb repressive complex 2 (PRC2)/AKT are known signaling mediators [122,123,124,125]. We previously discovered that ADT-mediated NED might be induced through an increase in ADT-induced ZBTB46, which triggers LIF expression [124]. Luo et al. reported that ADT altered AR-binding patterns in different androgen response elements (AREs), which caused the LncRNA-p21 expression. LncRNA-p21 alters the composition of PRC2, which methylates STAT3 and results in NED [126]. The PEDF is known to be a survival factor in neuron cells [127]. Smith et al. discovered that IL-6 triggered the ectopic expression of PEDF in PCa cells, and that expression reciprocally increased IL-6 expression via the RhoA/IKK/NF-κB axis [125]. When combined together, the aforementioned studies focus on the EMT and NED in STAT3 signaling in PCa, which can be summarized as STAT3 enabling immune escape and the EMT.

In addition to cell metamorphosis, STAT3 maintains PCa cell survival under ADT conditions. Previous studies have revealed that IL-6 and phospholipase Cε (PLCε) activated STAT3 via direct phosphorylation or non-canonical Hedgehog signaling that activates the AR, survivin, and NF-κB/promatrilysin expressions to maintain cell survival under ADT conditions [119,120,128,129,130]. In addition, the DAB2-interacting protein (*DAB2IP*) is a tumor-suppressor gene that blocks various survival-essential signaling pathways like PI3K/AKT, AR, JAK/STAT, and Wnt [131]. Zhou et al. found that DAB2IP loss in PCa could help cancer cells survive under ADT through STAT3-mediated survivin expressions [132]. By combining survival and the signaling network of STAT3-mediated EMT mentioned in the previous section, we have found that some overlap at IL6/STAT3 and STAT3/NF-κB as survival factors and NE inducers. However, Palmer, Hertzog, and Hammacher have reported that IL-6/STAT3 signaling only triggers NED, not cell growth [119]. That report carried a message that the switch between EMT/NED and cell survival/growth could not be simultaneously operated. The molecular details between STAT3-mediated EMT and cell growth still have large unfilled knowledge gaps. Furthermore, we discuss the role of EGFR and STAT3 signaling within CRPC-Adeno and CRPC-NE development that might be a survival keeper, AR repressor, or EMT inducer.

## 4. Interplay of EGFR and JAK/STAT3

**Figure 9:**
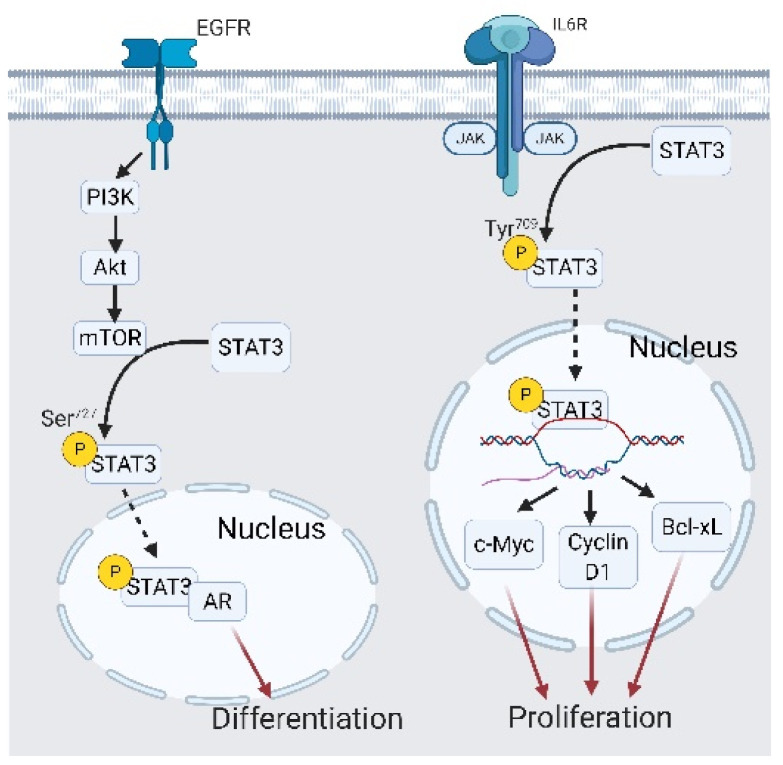
Interplay between the epidermal growth factor receptor (EGFR) and signal transduction and activator of transcription 3 (STAT3) in castration-resistant prostate cancer (CRPC) and CRPC-neuroendocrine (NE) development. EGFR/AKT/mammalian target of rapamycin (mTOR) signaling phosphorylates STAT3 at Ser^727^, which leads to STAT3 interacting with the androgen receptor (AR) binding with different androgen response elements, ultimately initiating differentiation. A black arrow represents signal transduction; a black dashed arrow represents translocated small molecules; a red arrow represents cell fate.

From the previous discussion, we have concluded that EGFR signaling helps to maintain PCa cell growth under ADT conditions, and that JAK/STAT3 signaling drives CRPC-Adeno cells into the EMT, which further fostered transdifferentiation into CRPC-NE. These two signaling pathways with distinct biological activities sometimes cooperate and trigger different cell outcomes in cancer cells. In fact, STAT3 is one of the downstream signaling transducers of EGFR signaling via the PI3K/AKT/mechanistic targets for the mammalian target of rapamycin (mTOR) cascade [133]. When activated by a cytokine receptor, non-receptor tyrosine kinases of cytokine receptors like JAK or Fer phosphorylate STAT3 at tyrosine 709 [133]. Y^709^-phosphorylated STAT3 is translocated into nuclei and initiates antiapoptotic and proliferation-related gene expressions such as *c-Myc*, *Bcl-xL*, and *cyclin-D1* [133,134,135]. When STAT3 is phosphorylated by PI3K/AKT/mTOR signaling at serine 727, STAT3 activity changes to an AR trans-activator that interacts with the N-terminal domain of the AR and promotes AR sensitivity toward androgen [136] (Figure 9). Moreover, STAT3/AR signaling tends to initiate differentiation instead of proliferation [133]. Collectively, EGFR is located upstream of STAT3 and modulates proliferation-related and differentiation-related STAT3 transcriptional targets. After this, we elaborate on a newly discovered EGFR/STAT3 crosstalk pattern that directly triggers castration resistance and subsequent NED.

**Figure 10:**
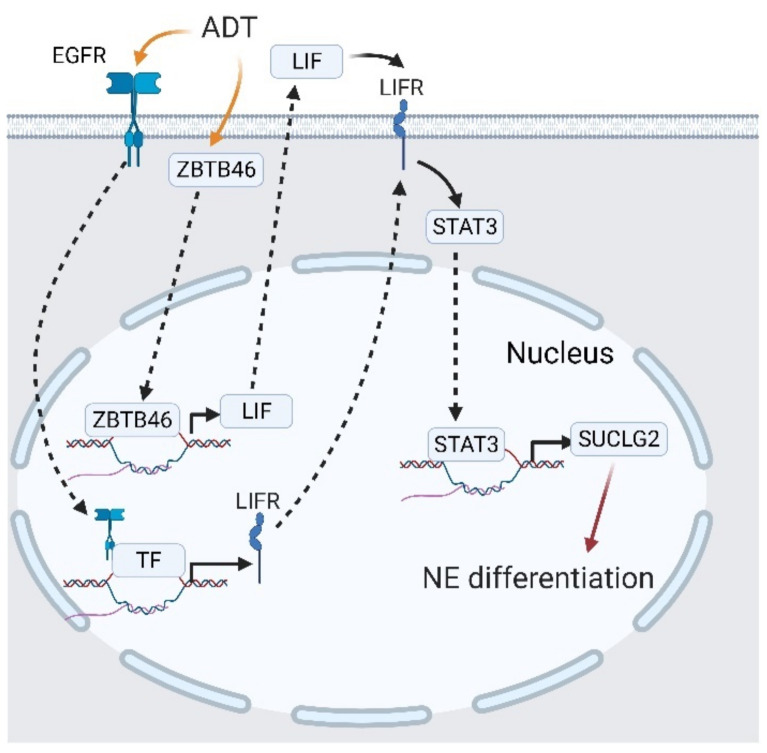
Interplay between the epidermal growth factor receptor (EGFR) and signal transduction and activator of transcription 3 (STAT3) in castration-resistant prostate cancer (CRPC) and CRPC-neuroendocrine (NE) development. Androgen deprivation therapy (ADT) stimulates EGFR translocation, followed by the leukemia-inhibitory factor receptor (LIFR) expression. The LIFR activates STAT3 and promotes SUCLG2 expression, which triggers neuroendocrine differentiation (NED). A black solid arrow represents signal transduction; a black dashed arrow represents translocation of proteins; a black faded arrow represents peptide hormones that bind to receptors; a red arrow shows the cell fate.

We have reported a novel mechanism of ADT-mediated CRPC-NE development, facilitated by EGFR translocation and STAT3 activation [137]. As PCa cells undergo ADT, the activated EGFR is translocated into nuclei and initiates LIF receptor (LIFR) expression. Upregulated LIFR promotes succinate-CoA ligase guanosine 5′-diphosphate (GDP)-forming beta subunit (SUCLG2) and finally induces NED [137]. The ectopic distribution of the EGFR is an unfavorable marker in various cancers including lung cancer, breast cancer, cervical cancer, and head and neck squamous cell carcinoma, which is related to radiotherapy resistance and dysregulation of the intracellular reduction/oxidation (ReDox) state [86,138,139,140]. In PCa, nuclear EGFR inhibits miR-1 expression, which is a suppressor of Twist1, and pushes PCa cells to undergo bone metastasis [141]. Moreover, nuclear EGFR can be found in CRPC cells from plasma membrane trafficking or neighboring exogenous vesicles, which implies a correlation between castration resistance and nuclear EGFR trafficking [90,142]. This discovery regarding nuclear EGFR/LIFR/SUCLG2 in CRPC-NE development confirmed the hypothesis in castration resistance and nuclear EGFR, which extends the correlation of nuclear EGFR with CRPC-NE development. By combining another report regarding ADT-mediated LIF upregulation promoting NED [124], we demonstrated a perspective that shows how ADT induces castration resistance and NE development. As PCa cells face ADT, some EGFR from plasma membranes are translocated into nuclei and initiates LIFR expression. Additionally, ADT also upregulates ZBTB46 expressions, which augments LIF expressions and promotes LIFR/STAT3 signaling in an autocrine or paracrine manner, induces SUCLG2 expression, and finally initiates NED (Figure 10).

## 5. Conclusions and Future Remarks

### 5.1. EGFR/STAT3 Target Therapy in CRPC Therapy

We have shown that EGFR and STAT3 are important modulators of CRPC-Adeno and CRPC-NE development during ADT as either standalone entities or via crosstalk. EGFR changes STAT3 activity from AR promotion to AR repression. Moreover, EGFR is translocated into the nuclei and elevates STAT3 signaling via the augmentation of receptors and ligands for cytokine receptor signaling upstream of STAT3. This novel signaling pathway seems to be a target for CRPC therapy. EGFR inhibitors have been tested in CRPC treatment. Cetuximab, an FDA-approved EGFR-monoclonal antibody, which improves the outcome of metastatic CRPC with EGFR and phosphatase and tensin homolog(PTEN) overexpression [143]. Lapatinib and dacomitinib show in vivo growth inhibitory activity by targeting to ErbB2 [144]. Clinical trials using gefitinib and erlotinib in CRPC treatment exhibit limited therapeutic efficacy [145,146]. These studies reveal that EGFR target therapy is only effective in specific patient populations. Nevertheless, studies about targeting EGFR and STAT3 in glioblastoma [147], non-small-cell lung cancer [148,149,150], and pancreatic cancer [151] exhibit promising therapeutic efficacy (Table 1). Codony-Servat et al. reveal that some EGFR inhibitors stimulate STAT3 phosphorylation at tyrosine 705 and further trigger downstream signaling [152]. Zhang et al. demonstrated that STAT3 inhibitor napabucasin could inhibit prostate cancer growth [153]. That is, targeting both EGFR and STAT3 might have good therapeutic efficacy against CRPC.

### 5.2. Issues Still Unsolved

A newly discovered EGFR translocation mechanism elicited new advancements in CRPC development. However, many unanswered questions remain unresolved with regard to the regulation of EGFR/STAT3 interplay. One attractive aspect is switching the EGFR between proliferation and differentiation. From previous discussions, we found that either EGFR or STAT3 proliferation and differentiation signaling cannot coexist. Differentiation seems to be stress-dependent; as a result, EGFR- and STAT3-mediated survival signals inhibited the repression of differentiation [97,119,120]. What is the key switch that changes the direction of PCa cell fate from proliferation to differentiation? Another interesting point concerns nuclear EGFR-mediated NED. Studies have indicated that nuclear EGFR initiated NED through the upregulating of LIF/LIFR expressions, followed by STAT3 activation [124,137]. However, the previous section elucidated that STAT3 phosphorylation by a cytokine receptor occurred at Y^709^ and facilitated PCa cell proliferation, not differentiation [133]. Does nuclear EGFR interact with STAT3 and change cell fate? The third unsolved question involves proliferation and NED stimulation in the ADT-mediated increase in the LIF. Proliferation and differentiation antagonize the other so that concurrent proliferation and differentiation in LIF-increased PCa cells seem to conflict with what is known [154]. This aberrant phenomenon could be explained by the antagonism between P53 and STAT3, in which STAT3 can reduce P53 expressions as activated by the LIF [155]. Another possible explanation might be intratumor heterogeneity [156], because it indicates diverse expression profiles within a single tumor or even within cultured cells [157,158]. Such genetic heterogeneity might result in distinct responses to the LIF. These questions were derived from recent discoveries.

## Figures and Tables

**Table 1:** Studies targeting EGFR and STAT3 in cancer treatment.

Cancers	EGFR Inhibitors	STAT3 Inhibitors	Results	References
GBM	Afatinib	Pacritinib (JAK inhibitor)	Synergistically decrease GBM cell vibility	[147]
NSCLC	Erlotinib/Gefitinib	W2014-S	W2014-S sensitize NSCLC cells to erlotinib and gefitinib Reduce tumor growth in vivo	[148]
	Gefitinib	LL1	Sensitize NSCLC cell to gefitinib	[149]
	Lupeol	Lupeol	Increase NSCLC cell apoptosis. Suppress cell proliferation and colony formation	[150]
PC	Erlotinib	Alantolactone	Increase cell apoptosis Reduce cell migration	[151]

GBM, glioblastoma; NSCLC, non-small-cell lung cancer; PC, pancreatic cancer.

## Data Availability

Not applicable.
